# Whole-Body Movements Increase Arm Use Outcomes of Wrist-Worn Accelerometers in Stroke Patients

**DOI:** 10.3390/s21134353

**Published:** 2021-06-25

**Authors:** Gerrit Ruben Hendrik Regterschot, Ruud W. Selles, Gerard M. Ribbers, Johannes B. J. Bussmann

**Affiliations:** 1 Department of Rehabilitation Medicine, Erasmus University Medical Center Rotterdam, P.O. Box 2040, 3000 CA Rotterdam, The Netherlands; r.selles@erasmusmc.nl (R.W.S.); g.ribbers@erasmusmc.nl (G.M.R.); j.b.j.bussmann@erasmusmc.nl (J.B.J.B.); 2 Department of Plastic and Reconstructive Surgery, Erasmus University Medical Center Rotterdam, P.O. Box 2040, 3000 CA Rotterdam, The Netherlands; 3 Rijndam Rehabilitation, Westersingel 300, 3015 LJ Rotterdam, The Netherlands

**Keywords:** stroke, upper extremity, arm use, upper limb performance, accelerometer, sensor, walking, rehabilitation

## Abstract

Wrist-worn accelerometers are often applied to measure arm use after stroke. They measure arm movements during all activities, including whole-body movements, such as walking. Whole-body movements may influence clinimetric properties of arm use measurements—however, this has not yet been examined. This study investigates to what extent arm use measurements with wrist-worn accelerometers are affected by whole-body movements. Assuming that arm movements during whole-body movements are non-functional, we quantify the effect of whole-body movements by comparing two methods: Arm use measured with wrist-worn accelerometers during all whole-body postures and movements (P&M method), and during sitting/standing only (sit/stand method). We have performed a longitudinal observational cohort study with measurements in 33 stroke patients during weeks 3, 12, and 26 poststroke. The P&M method shows higher daily paretic arm use outcomes than the sit/stand method (*p* < 0.001), the mean difference increased from 31% at week three to 41% at week 26 (*p* < 0.001). Differences in daily paretic arm use between methods are strongly related to daily walking time (r = 0.83–0.92). Changes in the difference between methods are strongly related to changes in daily walking time (r = 0.89). We show that not correcting arm use measurements for whole-body movements substantially increases arm use outcomes, thereby threatening the validity of arm use outcomes and measured arm use changes.

## 1. Introduction

In approximately 80% of the cases, a stroke leads to impairments in arm function in terms of muscle strength, voluntary control, coordination, and range of motion [1]. In-clinic assessment of arm function after stroke is often assumed to indicate arm use in daily life, i.e., the activities a person does with the arm in daily life. However, studies indicate that arm function and arm use are different constructs and need to be measured separately after stroke [2].

Wrist-worn accelerometers are often applied to measure arm use after stroke. For example, wrist-worn accelerometers have been used to assess arm use during rehabilitation poststroke [3,4,5], and to compare arm use levels between stroke patients and healthy subjects [6,7]. Wrist-worn accelerometers have also been applied to compare the arm use levels between the paretic and nonparetic arm after stroke [6,8]. Furthermore, studies explored the relationship between paretic arm use measured with wrist-worn accelerometers, arm function, and arm capacity after stroke [3,9]. Moreover, the change in arm use after stroke measured with wrist-worn accelerometers and the potential moderating role of psychological factors have been investigated [4].

Wrist-worn accelerometers measure arm use by recording the movements of the arms during all daily activities [3,4,5,7,9,10,11,12]. Thus, arm movements due to whole-body movements (e.g., walking, cycling, wheelchair transport, vehicle transport) influence arm use measurements with wrist-worn accelerometers [13,14]. However, arm movements due to whole-body movements are conceptually different from arm use during activities as eating with knife and fork, combing hair, and drinking. Arm movements due to whole-body movements are primarily non-functional, and therefore, they should ideally not be recorded as arm use. Quantifying non-functional arm movements due to whole-body movements as part of functional arm use is potentially problematic, since it may affect the clinimetric properties (e.g., validity, sensitivity to change, reliability) of arm use measurements with wrist-worn accelerometers [13]. For instance, when a patient walks, the non-functional arm movements due to walking will be measured as arm use by wrist-worn accelerometers, which may significantly increase the arm use outcomes of wrist-worn accelerometers, thereby reducing the validity of the arm use measurements. Similarly, an increase in the daily amount of walking after stroke may influence the change in daily arm use measured with wrist-worn accelerometers, thereby threatening the validity of the measured arm use changes.

These potential problems have been identified by previous studies, and different methods have already been proposed to correct arm use measurements with wrist-worn accelerometers for the effect of whole-body movements. These methods are measuring arm movements during sitting and standing [13,15], or calculating a ratio outcome between arms [14]. However, these methods are not widely adopted in the research field, because (1) they require more complex sensor set-ups and/or signal analyses, and (2) the effect of whole-body movements on arm use measurements with wrist-worn accelerometers is still unclear and has not yet been quantified. Most studies report arm use measurements with wrist-worn accelerometers without correcting for the effect of whole-body movements, which may affect the clinimetric properties of arm use outcomes. Therefore, studies quantifying the effect of whole-body movements on cross-sectional and longitudinal arm use measurements with wrist-worn accelerometers are urgently needed to determine the necessity of correcting arm use measurements regarding the effect of whole-body movements.

The present study quantifies the effect of whole-body movements on cross-sectional and longitudinal arm use measurements with wrist-worn accelerometers after stroke. Assuming that all arm movements during whole-body movements are non-functional, we quantified the effect of whole-body movements by comparing the arm use outcomes of two measurement methods: (1) Arm use outcomes measured with wrist-worn accelerometers during all whole-body postures and movements (P&M method), and (2) arm use outcomes measured with wrist-worn accelerometers during only sitting and standing periods (sit/stand method) [13,15]. The difference between the arm use outcomes of these two methods is the effect of whole-body movements on arm use measurements with wrist-worn accelerometers. We hypothesized that (1) whole-body movements, especially walking, increase arm use outcomes of wrist-worn accelerometers and the size of the effect depends on the amount of walking, and (2) the positive effect of walking on arm use measurements with wrist-worn accelerometers increases with time poststroke as a result of an increase in the daily amount of walking after stroke.

## 2. Materials and Methods

### 2.1. Participants

The present study was a longitudinal observational cohort study and part of another study investigating the change in objectively measured arm use during the first six months after stroke [15]. When designing and reporting the present study, we followed the STROBE recommendations for observational studies [16]. In the present study, we aimed to include at least 28 stroke patients, since this sample size can detect a medium effect (Cohen’s d = 0.50) with an alpha of 0.05 and a power of 0.80. Included were patients admitted to Rijndam Rehabilitation (Rotterdam, The Netherlands) after an ischemic or hemorrhagic stroke that suffered from a paretic arm or leg (defined as National Institutes of Health Stroke Scale (NIHSS) 5A/B or 6A/B 4 ≥ score > 0). Inclusion criteria were (i) 18 years or older, (ii) Mini-Mental State Examination (MMSE) >19, (iii) able to sit at least 30 min with back support. Excluded were patients who were more than three weeks after stroke when admitted to the rehabilitation clinic. The study was performed between September 2016 and September 2019. The study was conducted in accordance with the Declaration of Helsinki. The study was approved by the Medical Ethics Committee of Erasmus MC University Medical Center Rotterdam in The Netherlands (MEC-2015-687), and all participants provided written informed consent.

### 2.2. Procedures

A researcher performed arm use measurements at 3 weeks, 12 weeks, and 26 weeks poststroke, assessed arm function (Fugl-Meyer upper extremity assessment) and stroke severity (National Institutes of Health Stroke Scale (NIHSS) [17,18]) and collected demographic data. At three weeks after stroke, all patients were inpatient at Rijndam Rehabilitation and received standard poststroke treatment. At Rijndam Rehabilitation, the arm-hand therapy after stroke consists of the Concise Arm and Hand Rehabilitation Approach in Stroke (CARAS) [19,20]. At week 12 poststroke, some individuals were still at the rehabilitation center, while at week 26 poststroke, all patients were at home. The arm use measurements at home were performed by the same researcher.

### 2.3. Arm Use Measurements

**Figure 1:**
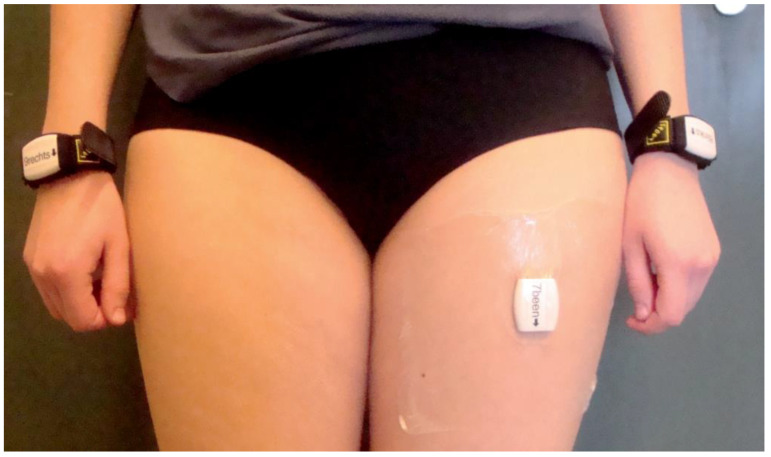
Participants wore three accelerometers: One accelerometer on each wrist and one accelerometer on the upper leg of the nonparetic side of the body.

In this study, we used an arm use monitor developed and validated for the measurement of arm use in stroke patients [13]. The arm use monitor consists of three accelerometers (Activ8 Activity Monitor, Activ8; 30 × 32 × 10 mm; 20 g). One accelerometer was attached to each wrist to measure arm movement intensity (see Figure 1), and one accelerometer was attached to the front side of the nonparetic thigh to recognize body postures and movements (lying, sitting, standing, walking, cycling, running). The applied accelerometers measured with a sampling frequency of 12.5 Hz [13]. The sensors on the wrists converted acceleration data to movement counts with 1.6 Hz resolution [13], and stored these data in epochs of 30 s (per epoch 48 samples). The sensor on the thigh converted acceleration data to movement counts and body postures/movements with 1.6 Hz resolution [13], and stored these data in epochs of 30 s (per epoch 48 samples). The recognition of body postures and movements (lying, sitting, standing, walking, cycling, running) by the sensor on the thigh is based on (1) the orientation of the sensor compared to gravity, and (2) the intensity of the movement (in movement counts) [13,21]. An Activ8 sensor on the thigh provides an accurate recognition of whole-body postures and movements in stroke patients with an accuracy ranging from 82 to 100% [21].

During weeks 3, 12, and 26 poststroke, patients wore the three accelerometers for seven consecutive days. The wrist-worn sensors were attached with wristbands and were taken off during the night and during water activities (e.g., showering, swimming). The leg sensor was worn for seven consecutive days and attached with anti-allergic, water-resistant skin tape. The data of the sensors were downloaded on a PC for further processing and analysis after each measurement period of one week.

### 2.4. Analysis of Sensor Data

To process and analyze the sensor data, we developed an algorithm in R [22] using RStudio (version 1.2.50001, RStudio, Inc., Boston, MA, USA). Firstly, the data of the sensors were time-synchronized based on the timestamps. We only analyzed waking hours from 7 a.m. to 10 p.m. Non-wear periods were excluded from further analysis and were defined as zero movement counts measured for at least one hour. Per participant, a measurement week was included in the analysis when at least two valid measurement days were available. A valid measurement day was defined as a day with at least ten hours of data of the whole sensor configuration.

In this study, we assumed that all arm movements during whole-body movements (e.g., walking, cycling, wheelchair transport, vehicle transport) are non-functional and conceptually different from arm use (e.g., combing hair, drinking, tooth brushing). Based on this assumption, we quantified the effect of whole-body movements on arm use measurements with wrist-worn accelerometers by comparing the arm use outcomes of two measurement methods: (1) Arm use measured with wrist-worn accelerometers during all whole-body postures and movements (P&M method), and (2) arm use measured with wrist-worn accelerometers during only sitting and standing periods (sit/stand method) [13,15]. The difference between the arm use outcomes of these two methods is the effect of whole-body movements on arm use measurements with wrist-worn accelerometers. Per valid measurement day, we calculated the arm use outcomes described below.

**P&M method:**

1. Paretic arm use: Calculated by summing the movement counts of the sensor on the paretic arm over all 30 s epochs.
2. Ratio between arms: Calculated as the paretic arm use during all whole-body postures and movements divided by the nonparetic arm use during all whole-body postures and movements.
3. Nonparetic arm use: Calculated by summing the movement counts of the sensor on the nonparetic arm over all 30 s epochs.

**Sit/stand method:**

1. Paretic arm use: Calculated by summing the movement counts of the sensor on the paretic arm over all 30 s epochs of which the posture was sitting or standing. An epoch was classified as sitting or standing when at least 90% of the 48 samples of the leg sensor were classified as sitting or standing.
2. Ratio between arms: Calculated as the paretic arm use during sitting and standing divided by the nonparetic arm use during sitting and standing.
3. Nonparetic arm use: Calculated by summing the movement counts of the sensor on the nonparetic arm over all 30 s epochs classified as sitting or standing.

Next, for each week (weeks 3, 12, and 26 poststroke), we calculated a mean daily value for each arm use outcome measure by averaging over valid measurement days.

### 2.5. Statistical Analysis

We performed the statistical analysis in R [22] using RStudio (version 1.2.50001, RStudio, Inc., Boston, MA, USA).

Differences in arm use outcomes between the P&M method and the sit/stand method were investigated at all time points (week 3, week 12, week 26 poststroke) by using Bland and Altman plots [23]. For the Bland and Altman plots, we calculated the mean difference in arm use outcome between the two methods (D), the SD of the differences in arm use outcome between the two methods (SDdiff), and the limits of agreement (LOA) as: LOA = D ± 1.96*SDdiff.

We applied Generalized Estimating Equation (GEE) [24] to test whether the P&M method and the sit/stand method differ significantly in cross-sectional and longitudinal arm use outcomes. In the GEE analysis, we included time (three levels: 3, 12, and 26 weeks), method (two levels: P&M method, sit/stand method), and the interaction time*method as factors. We used the Generalized Estimating Equation package (‘geepack’ package [25]) with as settings a Gaussian data distribution and an exchangeable correlation structure. Statistical significance was set at *p* < 0.05. For significant effects, posthoc comparisons were performed using the Estimated Marginal Means package (‘emmeans’ package) and by applying a Bonferroni correction [26].

To investigate whether differences in arm use outcomes between the methods are related to walking, we calculated Spearman’s rank correlation coefficients between the daily walking time and the difference in arm use outcome between the methods at each time point (week 3, week 12, week 26 poststroke). To examine whether changes in the daily amount of walking after stroke are related to differences in longitudinal arm use outcomes between the methods, we calculated Spearman’s rank correlation coefficients between the change in daily walking time from week 3 to week 26 poststroke and the change in arm use outcome difference between the methods from week 3 to week 26 poststroke. Correlations were interpreted as follows: Very weak when 0.00 < r < 0.25; weak when 0.25 ≤ r ≤ 0.49; moderate when 0.50 ≤ r ≤ 0.69; strong when 0.70 ≤ r ≤ 0.89; very strong when 0.90 ≤ r ≤ 1.00 [27].

## 3. Results

We included 33 stroke patients (26 males, 7 females). Table 1 shows the patient characteristics. The arm use data of three measurement weeks (weeks 3, 12, 26) were available from 18 patients, while from the other patients’ arm use data of only two measurement weeks were available. At week three poststroke, arm use data were missing in three participants as a result of a technical failure of the sensor system or non-wear of the system. At week 12 poststroke, arm use data were missing in five patients, due to a technical failure of the sensor system, non-wear of the system, or participant unavailability for the measurement. Arm use data were missing at week 26 in seven participants because of study dropout, a technical failure, or non-wear of the sensor system.

**Figure 2:**
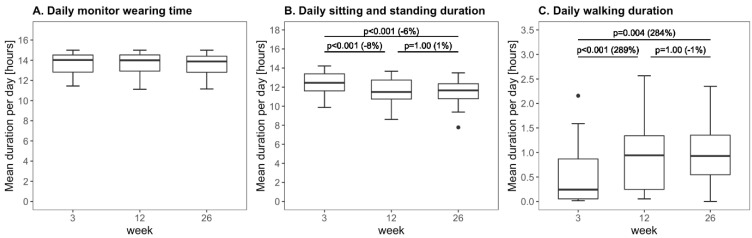
Boxplots of the daily monitor wearing time, daily sitting, and standing time, and daily walking time measured with the sensor system. The percentage between brackets represents the change in median value between time points.

The daily monitor wearing time did not change over time (*p* = 0.73; Figure 2A). Daily sitting and standing time decreased from week 3 to 12 and from week 3 to 26 (Figure 2B). Daily walking time increased from week 3 to 12 and from week 3 to 26 (Figure 2C).

**Figure 3:**
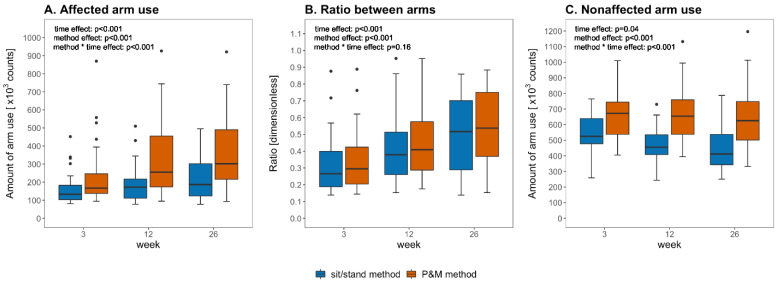
Boxplots showing the arm use outcomes of the P&M method and the sit/stand method at week 3, 12, and 26 poststroke. *p*-values of the GEE analyses are included in the plots.

**Figure 4:**
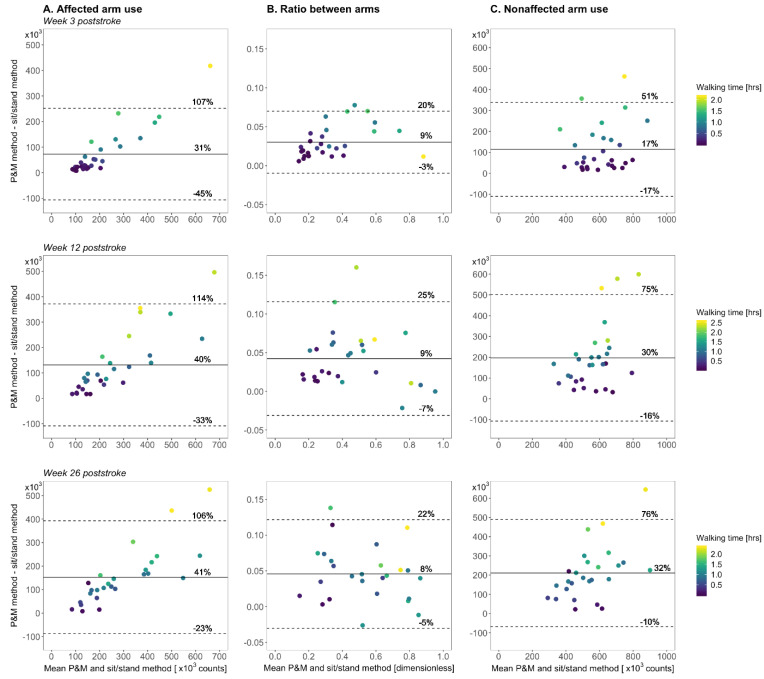
Bland and Altman plots showing the mean arm use outcome of the P&M method and the sit/stand method versus the difference in arm use outcome between the methods. The horizontal line indicates the mean difference between methods (D), and the dashed horizontal lines represent the limits of agreement (LOA). The D and LOA are expressed as a percentage of the mean arm use outcome of the P&M method. Bland and Altman plots are shown for week 3 poststroke (upper row), week 12 poststroke (middle row), and week 26 poststroke.

The P&M method showed higher paretic arm use, the ratio between arms, and nonparetic arm use outcomes than the sit/stand method at all time points (*p* < 0.001; Figure 3 and Figure 4). The mean difference in paretic arm use outcome between the methods increased over time (*p* < 0.001) from 31% at week 3 to 40% at week 12 and 41% at week 26 poststroke (Figure 4). The mean difference in ratio outcome between the methods did not change over time (*p* = 0.16) and was 8–9% at the different time points (Figure 4). The mean difference in nonparetic arm use outcome between the methods increased over time (*p* < 0.001) from 17% at week 3 to 30% at week 12 and 32% at week 26 poststroke (Figure 4).

**Figure 5:**
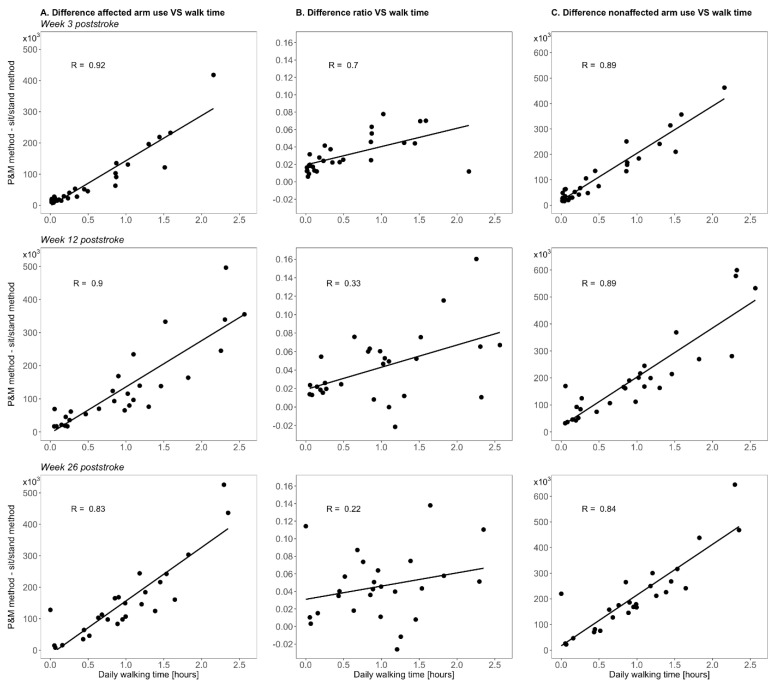
Scatterplots showing the daily walking time versus the difference in arm use outcome between the P&M method and the sit/stand method. Scatterplots are shown for week 3 poststroke (upper row), week 12 poststroke (middle row), and week 26 poststroke.

The differences in paretic and nonparetic arm use outcomes between the methods were strongly related to very strongly related to the daily walking time at all time points (r = 0.83–0.92; Figure 5), indicating a significant positive effect of walking on cross-sectional arm use measurements with the P&M method. The difference in ratio outcomes between the methods and the daily walking time were strongly related at week 3 poststroke (r = 0.70; Figure 5), but very weakly to weakly related at week 12 and week 26 poststroke (r = 0.22–0.33; Figure 5).

**Figure 6:**
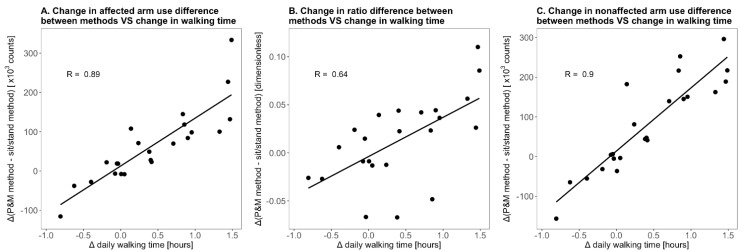
Scatterplots showing the change in daily walking time from week 3 to week 26 poststroke versus the change in arm use outcome difference between the P&M method and the sit/stand method from week 3 to week 26 poststroke.

The increase in paretic and nonparetic arm use differences between the methods from week 3 to week 26 was strongly related to very strongly related to the increase in daily walking time poststroke (r = 0.89–0.90; Figure 6), indicating a significant positive effect of walking on longitudinal arm use measurements with the P&M method. The change in the ratio differences between the methods from week 3 to week 26 was moderately related to the change in daily walking time (r = 0.64; Figure 6).

## 4. Discussion

Results of this study confirm our hypotheses by showing that whole-body movements increase cross-sectionally measured arm use outcomes of wrist-worn accelerometers by 8–41% if arm use data are not corrected for whole-body movements. We found that the size of the effect of whole-body movements on arm use measurements depends largely on the amount of walking. Since the daily amount of walking increased from week 3 to week 26 after stroke, the average effect of whole-body movements on paretic arm use outcomes increased from 31% at week 3 to 41% at week 26 poststroke when not correcting for whole-body movements. These findings indicate that not correcting arm use data for whole-body movements may threaten the validity of arm use outcomes and of measured changes in arm use over time.

The positive effect of walking on arm use measurements with wrist-worn accelerometers can be explained by the fact that wrist-worn accelerometers measure arm use by recording all arm movements. This includes non-functional arm movements as a result of the center of mass displacement during walking, which are measured as arm use by wrist-worn accelerometers, and which consequently increase the arm use outcomes of wrist-worn accelerometers. Since most patients increased in daily walking time from week 3 to week 26 poststroke (Figure 2), the positive effect of walking on paretic and nonparetic arm use measurements increased from week 3 to week 26 after stroke.

The positive effect of walking on the ratio between arms is less clear. Our data suggest that walking has a larger absolute effect on nonparetic arm use outcomes than on paretic arm use outcomes (Figure 4A,C)—possibly because of more arm sway of the nonparetic arm during walking—and that as a result, the ratio between arms is only slightly higher during walking than during sitting/standing (Figure 4B). This would explain (1) why the positive effect of whole-body movements on the ratio between arms did not substantially change over time (Figure 4B), (2) why changes in daily walking time were not strongly related to changes in the ratio difference between the two methods (Figure 6B), and (3) why daily walking duration showed relatively weak associations with the difference in ratio outcome between the methods (Figure 5B).

A noteworthy observation was that at all time points, the differences in paretic arm use outcomes between the P&M method, and the sit/stand method were larger at higher paretic arm use levels (Figure 4A). This can be explained by the fact that individuals with higher paretic arm use levels spend more time walking during the day (see colored data in Figure 4A), resulting in larger differences in paretic arm use outcomes between the two methods. The relationship that we found between paretic arm use levels and daily walking time is in line with other studies showing a relationship between the disability level of the paretic arm and walking performance after stroke [28].

Since whole-body movements, especially walking, greatly affect arm use measurements with wrist-worn accelerometers and threaten the validity of arm use outcomes, it is important to correct arm use measurements for this effect. The use ratio between arms was proposed by a previous study [14] to correct for the effect of whole-body movements. However, our results demonstrate that the ratio between arms cannot fully correct for the effect of whole-body movements, since whole-body movements increased the ratio between arms on average by 8–9% at all time points (Figure 4B). To correct arm use outcomes for the effect of whole-body movements, we propose to measure arm use by recording arm movements with wrist-worn accelerometers during only sitting and standing periods. This practical and simple method avoids the effect of walking and provides accurate arm use measurements in stroke patients [13]. The sensor configuration of this method currently consists of two wrist-worn accelerometers combined with an accelerometer on the upper leg to detect whole-body postures and movements. To foster the clinical application of this method, we are currently developing a minimal sensing solution by enabling the detection of whole-body postures and movements based on wrist-worn accelerometers instead of using an accelerometer on the upper leg.

Our study may have consequences for interpreting the results of other studies that did not correct arm use measurements for the effect of whole-body movements. The arm use outcomes of these studies may be affected by whole-body movements, especially walking. For example, a recent study applied wrist-worn accelerometers to measure arm use during the first 12 weeks after stroke without correcting for the effect of whole-body movements [4]. Results showed that the mean daily paretic arm use in 29 stroke patients increased by approximately 85% from about 2.6 h in week 2 poststroke to almost 5 h in week 12 after stroke. Since the study did not correct arm use measurements for whole-body movements, it is possible that whole-body movements, such as walking, have affected the reported arm use changes. This is plausible since we found a comparable (approximately 75%) increase in the mean daily paretic arm use from week 3 to week 12 after stroke when not correcting for whole-body movements, but a much smaller increase (19%) when correcting for whole-body movements. This example shows that whole-body movements may have affected the arm use outcomes of studies that did not correct for such an effect. A potential effect of whole-body movements should be taken into consideration when interpreting the findings of these studies.

Several limitations may have influenced the outcomes of this study. First, the relatively small sample size and the single recruitment site may limit the generalizability of our results. However, the sample size of the present study (n = 33) is larger than the required sample size (n = 28) that we estimated a priori based on a statistical power analysis (see Methods section). Second, at each time point in the study, there were missing data due to technical issues, non-wear of the system, or unavailability of participants. To avoid that missing data affected the outcomes of the study, we applied generalized estimating equations that can handle missing data appropriately [24]. Third, the accuracy of the detection of sitting, standing, and walking by the leg accelerometer is not perfect (approximately 90–95% [21]). However, since the detection accuracy is very high, it is unlikely that misclassification has affected the findings of the present study. Fourth, we did not consider the effect of dominance on the paretic arm use measurements, since previous research indicated that the difference in daily use between the dominant and non-dominant arm in healthy adults is very small [10]. Fifth, the analysis in the present study assumed that all arm movements during whole-body movements are non-functional. Although this assumption might not be fully correct, previous research has shown that measuring arm movements with wrist-worn accelerometers during only sitting and standing periods provides very accurate arm use outcomes in stroke patients [13], thereby supporting the validity of our assumption.

## 5. Conclusions

This study shows that whole-body movements increase cross-sectionally measured arm use outcomes of wrist-worn accelerometers with 8–41% if not correcting arm use data for whole-body movements. We found that the size of the effect of whole-body movements on arm use measurements depends largely on the amount of walking. Since the daily amount of walking increased from week 3 to week 26 poststroke, the average effect of whole-body movements on paretic arm use outcomes increased from 31% at week 3 to 41% at week 26 poststroke when not correcting for whole-body movements. These findings indicate that not correcting arm use data for whole-body movements may threaten the validity of arm use outcomes and of measured changes in arm use over time. To correct arm use measurements with wrist-worn accelerometers for the effect of whole-body movements and specifically walking, we propose a practical and valid solution that measures arm use during only sitting and standing periods with wrist-worn accelerometers and an accelerometer on the upper leg [13].

## Figures and Tables

**Table 1:** Patient characteristics (n = 33). Data are reported as mean ± SD [minimal value, maximal value] unless otherwise stated.

Age in Years	55.9 ± 9.2 [37–75]
Gender	26 males, 7 females
Affected body side	12 left side, 21 right side
Dominant side affected	11 (33%)
Admitted to rehabilitation clinic in weeks poststroke	1.6 ± 0.7 [0.4–3.0]
Discharge from a rehabilitation clinic in weeks poststroke	10.5 ± 4.7 [3.7–20.3]
NIHSS ^a^ values week 12 poststroke	2.1 ± 2.7 [0–11]
Fugl-Meyer upper extremity assessment:	
week 3 poststroke	25.5 ± 20.6 [4–64]
week 12 poststroke	41.2 ± 21.8 [4–64]
week 26 poststroke	51.2 ± 16.8 [9–64]

^a^ National Institutes of Health Stroke Scale.

## Data Availability

The data presented in this study are available on request from the corresponding author. The data are not publicly available due to privacy/ethical restrictions.
